# Economic Evaluations and Equity in the Use of Artificial Intelligence in Imaging Exams for Medical Diagnosis in People With Skin, Neurological, and Pulmonary Diseases: Protocol for a Systematic Review

**DOI:** 10.2196/48544

**Published:** 2023-12-28

**Authors:** Giulia Osório Santana, Rodrigo de Macedo Couto, Rafael Maffei Loureiro, Brunna Carolinne Rocha Silva Furriel, Edna Terezinha Rother, Joselisa Péres Queiroz de Paiva, Lucas Reis Correia

**Affiliations:** 1 PROADI-SUS Hospital Israelita Albert Einstein São Paulo Brazil; 2 Imaging Department Hospital Israelita Albert Einstein São Paulo Brazil; 3 Department of Preventive Medicine Universidade Federal de São Paulo São Paulo Brazil; 4 Computer Engineering School Universidade Federal de Goiás Goiânia Brazil; 5 Studies and Research in Science and Technology Group (GCITE) Instituto Federal de Goiás Goiânia Brazil; 6 Instituto Israelita de Ensino e Pesquisa Hospital Israelita Albert Einstein São Paulo Brazil; 7 Department of Preventive Medicine Universidade de São Paulo São Paulo Brazil

**Keywords:** artificial intelligence, economic evaluation, equity, medical diagnosis, health care system, technology, systematic review, cost-effectiveness, imaging exam, intervention

## Abstract

**Background:**

Traditional health care systems face long-standing challenges, including patient diversity, geographical disparities, and financial constraints. The emergence of artificial intelligence (AI) in health care offers solutions to these challenges. AI, a multidisciplinary field, enhances clinical decision-making. However, imbalanced AI models may enhance health disparities.

**Objective:**

This systematic review aims to investigate the economic performance and equity impact of AI in diagnostic imaging for skin, neurological, and pulmonary diseases. The research question is “To what extent does the use of AI in imaging exams for diagnosing skin, neurological, and pulmonary diseases result in improved economic outcomes, and does it promote equity in health care systems?”

**Methods:**

The study is a systematic review of economic and equity evaluations following PRISMA (Preferred Reporting Items for Systematic Reviews and Meta-Analyses) and CHEERS (Consolidated Health Economic Evaluation Reporting Standards) guidelines. Eligibility criteria include articles reporting on economic evaluations or equity considerations related to AI-based diagnostic imaging for specified diseases. Data will be collected from PubMed, Embase, Scopus, Web of Science, and reference lists. Data quality and transferability will be assessed according to CHEC (Consensus on Health Economic Criteria), EPHPP (Effective Public Health Practice Project), and Welte checklists.

**Results:**

This systematic review began in March 2023. The literature search identified 9,526 publications and, after full-text screening, 9 publications were included in the study. We plan to submit a manuscript to a peer-reviewed journal once it is finalized, with an expected completion date in January 2024.

**Conclusions:**

AI in diagnostic imaging offers potential benefits but also raises concerns about equity and economic impact. Bias in algorithms and disparities in access may hinder equitable outcomes. Evaluating the economic viability of AI applications is essential for resource allocation and affordability. Policy makers and health care stakeholders can benefit from this review’s insights to make informed decisions. Limitations, including study variability and publication bias, will be considered in the analysis. This systematic review will provide valuable insights into the economic and equity implications of AI in diagnostic imaging. It aims to inform evidence-based decision-making and contribute to more efficient and equitable health care systems.

**International Registered Report Identifier (IRRID):**

DERR1-10.2196/48544

## Introduction

Traditional health care systems encounter a multitude of challenges that have persisted for decades. These challenges include patient diversity, the vast geographical areas they are often required to cover, and the ever-present financial constraints that limit their capacity to deliver optimal care [1]. However, in recent years, the advent of artificial intelligence (AI) has introduced a transformative dimension to health care, showing immense potential in mitigating these challenges [2,3].

AI, a multidisciplinary field, integrates computer science and engineering to generate intelligent machines skilled in emulating human cognition and problem-solving [4]. Its subfield, machine learning, refines algorithms through the extensive analysis of data sets, thus enabling decision-making and predictions across various contexts [5,6]. An advanced offshoot, deep learning, has found particular prominence in medical imaging, bolstering the system’s capacity for human-like reasoning [7]. Within health care, AI technologies serve critical roles in enhancing the accuracy and efficiency of disease detection, segmentation, and classification in medical imaging [8]. These advancements contribute to improved clinical decision-making, cost reduction, and error mitigation [9].

Nevertheless, there are pressing concerns that must be urgently addressed to maximize the clinical use of AI in health care. Methodological limitations, such as the lack of transparent algorithms and insufficient validation studies, risk hindering the wider adoption and efficacy of AI systems in clinical settings [10,11]. Moreover, the issue of data set bias warrants immediate attention; many of the data sets currently used for training and validating AI models are inherently skewed toward specific ethnic or demographic groups [12]. This imbalance may result in algorithms that are less effective or even misleading when applied to populations not adequately represented in the training data, thereby perpetuating existing inequalities in health care outcomes [13]. Such weaknesses not only constrain the clinical impact but could also exacerbate existing health disparities by delivering suboptimal or biased care [14].

Health equity refers to the principle that everyone should have equal access to health care and the opportunity to attain the best possible health, regardless of personal characteristics such as race, ethnicity, gender, socioeconomic class, or other factors that can contribute to health disparities [15]. Moreover, it is crucial to acknowledge that even when data related to equity or social determinants of health are incorporated, they can inadvertently introduce biases into algorithms if not adequately integrated and interpreted [16]. Recently, AI has gained significant attention due to its equity implications, underscoring the imperative to promote population well-being while taking into account differences in gender, race, and socioeconomic status, as evidenced by multiple sources [17-19].

Brazil, a large upper-middle–income country, has the world’s largest free public health care system, which is relied upon by the majority of its population. However, several socioeconomic barriers hinder access to diagnoses in impoverished and remote areas. Digital health initiatives, including AI-powered clinical decision support systems, have proven effective in reducing health disparities and facilitating access to services [20]. To tackle these challenges, the Brazilian Ministry of Health’s Support Program for the Institutional Development of the Unified Health System (in Portuguese, “Programa de Apoio ao Desenvolvimento Institucional do Sistema Único de Saúde”, known as PROADI-SUS) supports a collaborative project named “Banco de Imagens” (Bank of Images) to create a nationwide cloud-based repository of medical images, as well as to develop and validate AI algorithms to assist in disease diagnosis. Currently, clinical validation is underway for performance assessment of diagnostic decision-support algorithms, specifically engineered to address conditions of Brazilian public health importance. These algorithms target dermatological lesions such as melanoma, radiological markers indicative of pulmonary tuberculosis, and volumetric alterations in cerebral computed tomography scans for the evaluation of conditions, including microcephaly, brain atrophy, and hydrocephaly. To ensure the effectiveness and accessibility of these AI algorithms, the clinical validation process also aims to measure the economic and equity impact of implementing these technologies in real-world.

Hence, this protocol outlines a systematic literature review that aims to investigate the economic performance or viability of using AI in diagnostic imaging exams, particularly for skin, neurological, and pulmonary diseases. The review will also assess the possible equity outcomes of this intervention in health care systems. The research question guiding this review is “To what extent does the use of AI in imaging exams for diagnosing skin, neurological, and pulmonary diseases result in improved economic outcomes, and does it promote equity in health care systems?” A preliminary search for existing systematic reviews on this topic has been conducted in databases such as PubMed and PROSPERO, and no identical review to the proposed study was found.

## Methods

### Overview

The study is designed as a systematic review of economic evaluations, which will be reported in accordance with the PRISMA-P (Preferred Reporting Items for Systematic Review and Meta-Analysis Protocols) checklist [21]. To ensure good reporting practices for economic evaluations, the PRISMA (Preferred Reporting Items for Systematic Reviews and Meta-Analyses) tool [22] will be used along with the CHEERS (Consolidated Health Economic Evaluation Reporting Standards) checklist [23]. Additionally, we will adhere to guidelines provided by Preferred Reporting Items for Systematic Reviews and Meta-Analyses with a Focus on Health Equity (PRISMA-E) for conducting equity-focused systematic reviews [24]. The research question was structured following the population, intervention, comparison, and outcomes structure, as shown in Table 1.

**Table 1:** Population, intervention, comparison, and outcomes (PICO) structure for the systematic review of global economic evaluations and equity studies in the use of artificial intelligence (AI) in medical diagnosis of people with skin, neurological, and pulmonary diseases.

Acronym	Description	Criteria
P	Population	Patients screened for pulmonary, neurological, and cutaneous diseases, considering the entire world population, regardless of the income and development of that country
I	Intervention	Use of AI for diagnostic decision support in any type of imaging exam
C	Comparison	Conventional or human-based diagnostic methods
O	Outcomes	Full economic evaluations, such as cost-effectiveness, cost-usefulness, cost-benefit, or cost-minimization. We will assess changes in patient outcomes related to health care access and demographic factors like age, gender, race, and income, with a focus on equity.

### Eligibility Criteria

Publications on economic evaluations or equity in diagnostic imaging use AI-based tools will be included. The thematic focus will align with the specialization areas of the Bank of Images project, particularly emphasizing conditions of critical importance to public health. These include (1) dermatology, specifically melanoma and skin carcinomas; (2) neurology, targeting disorders with notable radiological findings; and (3) pulmonology, concentrating on conditions such as tuberculosis, lung consolidation, pleural effusion, atelectasis, pneumothorax, mediastinal widening, lung edema, lung opacity, lung lesion, lung cancer, other pleural diseases, and lung nodules. Detailed inclusion and exclusion criteria, encompassing language and publication year, are provided in Textbox 1.

**Inclusion and exclusion criteria for the systematic review of global economic evaluations and equity studies in the use of artificial intelligence in medical diagnosis of people with skin, neurological, and pulmonary diseases.**

**Inclusion criteria**

- Article type: Original articles reporting economic evaluation or equity. Cost-effectiveness, cost-usefulness, or cost-benefit or cost-minimization analysis will be considered as economic evaluation studies. Regarding equity, the authors will analyze reported improvements or declines in patient outcomes based on access to health care and demographic indicators such as age, gender, race, and income level.
- Study object: Use of artificial intelligence–based tools in diagnostic imaging exams in dermatological, pulmonary, and neurological fields.
- Language: There will be no restrictions based on language.
- Year of publication: There will be no restrictions based on year of publication.

**Exclusion criteria**

- Article type: Partial economic evaluations, such as cost analysis, cost-description studies, and cost-outcome descriptions. Regarding equity, we will exclude studies that stratify the study sample into a homogeneous population, not representative of the general population.
- Study object: We will exclude studies that focus on other medical fields, such as heart diseases, breast cancer, and diabetes. Also, validation studies of artificial intelligence will be excluded.

### Information Sources

We will conduct a search in the PubMed, Embase, Scopus, and Web of Science databases and perform supplementary searches to locate references cited in the retrieved articles.

### Search Strategy

We will define a customized search strategy for each database, using terms related to AI, relevant diseases or health conditions as aforementioned, economic evaluations, and equity. Separate searches will be conducted for each field of medicine, as well as a general search without a specific field. The final search terms are provided in Tables S1-S4 in Multimedia Appendix 1.

### Data Management

We will use an Excel (Microsoft) spreadsheet to manage and organize records and data throughout the review process.

### Selection Process

The Rayyan platform (Qatar Computing Research Institute) will be used for article selection, with duplicates excluded by the platform and documented accordingly.

The selection process will consist of 2 phases. In the first phase, 2 researchers will independently evaluate and select articles. Researchers GOS and RML will evaluate titles and abstracts related to the neurological and pulmonary fields, while researchers GOS and BCRSF will assess titles and abstracts related to the dermatological field. Researchers GOS and RdMC will review titles and abstracts from the additional search that encompasses various fields of medicine.

In the second phase, the selected articles from the first phase will undergo full-text review. Researchers GOS and RML will evaluate full texts related to neurological and pulmonary fields, while researchers GOS and BCRSF will evaluate full texts related to the dermatological field. Researchers GOS and RdMC will review full texts resulting from the general search. Any disagreements will be resolved by a third researcher (RdMC for searches in neurological, pulmonary, and dermatological fields, and RML for the general search).

After the search strategy is completed, the authors will compile the final list of publications using EndNote software (version X9.3.3; Clarivate).

### Data Collection Process

After completing the article selection process, 2 researchers (GOS and RdMC) will collect the variables of interest for the systematic review from each publication with available data. A pilot form will be used to collect data from a subset of studies, following which data will be collected independently. The extracted data by each researcher will be compared and merged into a single Excel spreadsheet.

### Data Items, Outcomes, and Prioritization

List 1 registers the variables that will be collected from each study: ID, publication title, year of publication, publisher, authors, URL, type of study, language, time horizon, place of study, study population, study perspective, corresponding author’s contact, brief summary, objectives, methodology, sample size, statistical significance, applied AI, outcome measures, such as incremental cost-effectiveness ratio (ICER), cost-benefit, QALY (quality adjusted life years), applied model, sensitivity analysis, demographic characteristics of participants, health indicators of subpopulations, access to health services, measures of equity such as odds ratio and relative risk, direction of effect (favorable or not), first analysis of methodological quality, conflict of interest, funding, transferability of results, observations.

### Risk of Bias in Individual Studies and Transferability

The quality of the methodology evaluation will be assessed using the CHEC (Consensus on Health Economic Criteria) checklist [25], which includes a question on distributional effects to evaluate the equity aspects of economic evaluation studies. For selected studies that are not economic evaluations but were chosen for their equity results, the EPHPP (Effective Public Health Practice Project) quality assessment tool for quantitative studies [26] will be used to assess the risk of bias. The Welte checklist [27] will be used to evaluate the transferability.

For the CHEC checklist, points will be awarded for each of the met criteria, totaling a total of 20 items for evaluation. The point will not be awarded if the criterion is not fully met. A percentage value will be calculated based on the number of points awarded divided by the total points on the checklist multiplied by 100.

Regarding the EPHPP tool, each section will be assigned a rating of 1 (strong), 2 (moderate), or 3 (weak). At the end, a global score will be calculated according to the ratings evaluated for each section where, 1 when there is no section as weak, 2 when there is at least 1 weak section, and 3 when there are at least 2 or more weak sections.

For the Welte checklist, points will be awarded according to the following criteria: perspective (2 points), discount rate (2 points), addressing medical costs (2 points), absolute and relative prices in health care (1 point), clinical practice variability (1 point), incidence and prevalence (1 point), mix of cases (1 point), life expectancy (1 point), productivity and lost work time (1 point), making a total of 12 points. Studies that obtained a result greater than or equal to 10 points will be considered transferable.

### Data Synthesis

We will create summary tables that include descriptive data from the studies, such as sample size, methods, cost-effectiveness rates, and other results. Information related to the country and health care system, year of publication, as well as the quality of methods and potential conflicts of interest, will be used to categorize results. Relevant findings from the studies will be summarized in the text. The main outcomes that we will focus on are (1) ICER, a cost-effectiveness measure that compares 2 health interventions; (2) cost-benefit results, an economic evaluation that compares the costs and benefits of a technology expressed in monetary units; and (3) equity results for studies that report them.

The methodologies used in the studies will be analyzed, and their robustness will be discussed. To facilitate comparisons between studies, all reported currencies will be converted to US dollars and adjusted for inflation using the US Consumer Price Index (CPI).

### Meta-Bias

We will consider the potential for meta-bias resulting from the selective reporting of studies and outcomes, where negative results are often omitted from publication. To mitigate this issue, we will conduct funnel plot analyses to identify possible asymmetries, particularly in the context of cost-effectiveness outcomes. This is crucial as non–cost-effective results may be underreported in the literature.

## Results

This systematic review began in March 2023 and has been registered with PROSPERO under registration number CRD42023407755. The literature search identified 9,526 publications and, after full-text screening, 9 publications were included in the study. Data summarization is currently underway. We plan to submit a manuscript to a peer-reviewed journal once it is finalized, with an expected completion date in January 2024.

## Discussion

### Principal Findings

The systematic review presented in this protocol aims to address the use of AI in imaging exams for the diagnosis of skin, neurological, and pulmonary diseases. Considering the challenges faced by traditional health care systems, such as patient heterogeneity, geographic disparities, and financial constraints, the integration of AI technology offers a promising avenue to enhance health care delivery [1-3]. This systematic review seeks to synthesize existing evidence to cost-evaluate AI-driven diagnostic interventions and their potential impact on promoting equitable health care.

The inclusion of AI algorithms in health care has shown promise in various domains, particularly in medical imaging analysis. AI can assist in the detection, segmentation, and classification of medical images, potentially improving clinical decision-making and reducing diagnostic errors [8,9]. Moreover, AI has the potential to streamline health care processes, minimize costs, and facilitate earlier disease identification, which is of utmost importance in the context of skin, neurological, and pulmonary diseases [9].

Equity concerns in the implementation of AI-based tools for diagnostic imaging exams have gained significant attention in recent years. While these technological advancements hold immense promise for enhancing health care outcomes, they also carry the risk of exacerbating existing disparities in access to high-quality care. A major concern revolves around the potential for bias in AI algorithms, which could disproportionately affect marginalized and underrepresented patient groups. If AI systems are not adequately trained on diverse data sets, they may exhibit biases related to race, gender, or socioeconomic status, leading to inaccurate diagnoses or recommendations for specific demographic segments. Furthermore, the significant costs of adopting AI technologies could create disparities in access, favoring well-funded health care institutions over underresourced ones. Additionally, the digital divide, wherein not all communities are afforded equal access to essential technology and infrastructure, can further compound inequities in AI-supported diagnostic imaging. Addressing these challenges in an effective manner is crucial for the equitable distribution of AI’s benefits in health care, ensuring that they reach all patient demographics irrespective of their socioeconomic or cultural backgrounds [1-3].

The measurement of the economic impact of AI-based tools in diagnostic imaging holds profound significance for several reasons. First, a comprehensive understanding of this impact enables health care systems to allocate their resources more judiciously, ensuring that investments yield tangible benefits and lead to cost savings. Also, AI has the potential to optimize the use of resources and, in turn, reduce the overall expenses associated with health care delivery. Moreover, assessing the economic viability of AI tools plays a pivotal role in determining their affordability and accessibility. By discerning their cost-effectiveness, it becomes feasible to identify potential obstacles to access and devise strategies that guarantee the availability of state-of-the-art health care to all individuals, regardless of their income or geographic location. Finally, identifying AI applications with the most promising economic potential can guide investment decisions, foster innovation, and expedite the development of tools that have a positive impact on patient care.

As with any systematic review, certain limitations are anticipated. One potential limitation is the availability and quality of the included studies. Variability in study designs, data sources, and methodological approaches may present challenges in synthesizing findings and drawing conclusive results. Regarding the topic of AI, as it is a recent object of study and because terms are delimited into 3 medical areas (dermatological, neurological, and pulmonary), there may be a shortage of available primary studies. This can limit the ability to perform robust statistical analyses or draw definitive conclusions. Publication bias is also recognized, in which researchers tend to publish only positive results. Furthermore, some technologies developed in specific contexts (eg, high-income or White-majority countries) may not be applicable in other realities or countries.

### Conclusions

The findings of this systematic review will hold significant implications for policy makers, health care practitioners, and researchers. By comprehensively evaluating the economic performance and equity outcomes of AI use in diagnostic imaging for skin, neurological, and pulmonary diseases, we aim to provide valuable insights into the integration of AI into clinical practice. These insights can inform evidence-based decision-making, potentially leading to more efficient and equitable health care systems.

## Appendix

Multimedia Appendix 1 Search strategy.

## Abbreviations

AI: artificial intelligence
CHEC: Consensus on Health Economic Criteria
CHEERS: Consolidated Health Economic Evaluation Reporting Standards
CPI: Consumer Price Index
EPHPP: Effective Public Health Practice Project
ICER: incremental cost-effectiveness ratio
PRISMA: Preferred Reporting Items for Systematic Reviews and Meta-Analyses
PRISMA-E: Preferred Reporting Items for Systematic Reviews and Meta-Analyses with a Focus on Health Equity
PRISMA-P: Preferred Reporting Items for Systematic Review and Meta-Analysis Protocols
QALY: quality adjusted life years

## Data Availability

All data generated or analyzed during this study are included in this published article and its supplementary information files.
